# Rehabilitation of hand function after spinal cord injury using a novel handgrip device: a pilot study

**DOI:** 10.1186/s12984-017-0234-1

**Published:** 2017-03-21

**Authors:** Haydn Hoffman, Tiffany Sierro, Tianyi Niu, Melanie E. Sarino, Majid Sarrafzadeh, David McArthur, V. Reggie Edgerton, Daniel C. Lu

**Affiliations:** 1 Department of Neurosurgery, University of California, Los Angeles, 300 Stein Plaza, Ste. 536, Los Angeles, CA 90095-6901 USA; 2 Rancho Los Amigos National Rehabilitation Center, Downey, CA 90242 USA; 3 Wireless Health Institute, University of California Los Angeles, Los Angeles, CA USA; 4 Department of Computer Science, University of California Los Angeles, Los Angeles, CA USA; 5 Department of Orthopedic Surgery, David Geffen School of Medicine, University of California, Los Angeles, Los Angeles, CA 90095 USA; 6 Neuromotor Recovery and Rehabilitation Center, David Geffen School of Medicine, University of California, Los Angeles, Los Angeles, CA 90095 USA; 7 Brain Research Institute, University of California, Los Angeles, Los Angeles, CA 90095 USA

**Keywords:** Spinal cord injury, Hand function, Neurorehabilitation, Activity-based therapy

## Abstract

**Background:**

Activity-based therapy (ABT) for patients with spinal cord injury (SCI), which consists of repetitive use of muscles above and below the spinal lesion, improves locomotion and arm strength. Less data has been published regarding its effects on hand function. We sought to evaluate the effects of a weekly hand-focused therapy program using a novel handgrip device on grip strength and hand function in a SCI cohort.

**Methods:**

Patients with SCI were enrolled in a weekly program that involved activities with the MediSens (Los Angeles, CA) handgrip. These included maximum voluntary contraction (MVC) and a tracking task that required each subject to adjust his/her grip strength according to a pattern displayed on a computer screen. For the latter, performance was measured as mean absolute accuracy (MAA). The Spinal Cord Independence Measure (SCIM) was used to measure each subject’s independence prior to and after therapy.

**Results:**

Seventeen patients completed the program with average participation duration of 21.3 weeks. The cohort included patients with American Spinal Injury Association (ASIA) Impairment Scale (AIS) A (*n* = 12), AIS B (*n* = 1), AIS C (*n* = 2), and AIS D (*n* = 2) injuries. The average MVC for the cohort increased from 4.1 N to 21.2 N over 20 weeks, but did not reach statistical significance. The average MAA for the cohort increased from 9.01 to 21.7% at the end of the study (*p* = .02). The cohort’s average SCIM at the end of the study was unchanged compared to baseline.

**Conclusions:**

A weekly handgrip-based ABT program is feasible and efficacious at increasing hand task performance in subjects with SCI.

## Background

Spinal cord injury (SCI) results in irreparable damage to spinal pathways [1], but some degree of functional recovery is often attainable [2]. Expanding this recovery has been the aim of rehabilitation. Previously, strategies for improving independence and quality of life among SCI patients focused on compensatory strategies utilizing muscles not affected by the spinal cord lesion [3]. This approach has changed in response to studies demonstrating neurologic improvement from activity-based therapies (ABT) that involve repetitive use of the affected muscle groups through exercise, somatosensory stimulation, and task-specific training [4–7]. ABT includes patterned or non-patterned motor activation as well as sensory stimulation [8]. Previous applications to SCI patients have not focused on hand-specific training or quantitative measurements of hand function. However, task-oriented ABT has been shown to improve hand function and performance of activities of daily living (ADLs) in stroke rehabilitation [9, 10]. ABT targeting hand function may have similar efficacy in SCI patients but has not been adequately investigated.

Loss of hand function is a particularly devastating aspect of SCI that patients associate with a reduction in quality of life (QoL) [11]. Exercise-based therapies that aim to increase recruitment of muscles above and below the affected spinal level and to train the patient to perform specific tasks have enhanced neurologic recovery of the upper extremity in SCI [12, 13]. Subjects with chronic SCI and AIS A or B injuries have shown improvements in ASIA motor scores after completing 6 months of an intense, structured exercise program [14]. Furthermore, significant increases in independence have been demonstrated in patients with motor complete SCI after 1 year of rehabilitation [2]. The efficacy of these interventions merit investigation of their applicability to hand function.

The goal of this pilot study was to evaluate the efficacy and feasibility of a weekly hand testing protocol that emphasizes recruitment, strengthening, and task performance with a novel handgrip device that tracks grip force continuously. We sought to determine whether or not this approach would improve hand strength, task performance, and independence in performing daily activities. The handgrip was utilized because it allowed us to conduct reproducible, standardized therapy sessions that could be adapted according to each subject’s grip strength. Furthermore, it provided sensitive measurements of hand contraction and task performance. A portable device with these functions has not been described for SCI patients. Results of this study could help tailor future approaches towards ABT by elucidating the duration, type, and extent of activity required to yield a response among SCI patients. It will also serve as a pilot study for a larger scale application of the handgrip device.

## Methods

### Patient selection

Patients with cervical SCI were recruited for an NIH-funded clinical trial. They were referred for this study by their primary care physicians or neurologists, who were not part of this research study. From June 2014 to September 2014, 18 consecutive chronic, stable cervical spinal cord injured subjects, who met the inclusion and exclusion criteria were recruited from the clinic. The inclusion criteria were chronic stable spinal cord injury (>12 months from time of injury), age 18–60, and American Spinal Injury Association score A-D. The subjects were excluded from the study if they had any of the following comorbidites: major psychiatric illness, cardiac disease, diabetes, hypertension, body mass index > 25, cardiac pacemaker, implanted defibrillators, or existing implanted neurostimulators. Each subject’s eligibility was carefully reviewed by the senior author (DCL) after a clinic appointment, and each patient’s medical record and diagnostic images were reviewed. “Motor complete” injuries are defined as ASIA Impairment Scale (AIS) A or B injuries, while “motor incomplete” refers to AIS C or D.

### Intervention

**Fig. 1:**
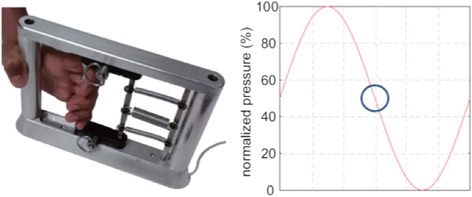
The handgrip device used to detect maximum voluntary contraction (*left*) and the sinusoidal tracking pattern that was displayed for the subjects to follow by adjusting grip strength (*right*). Figure adapted with permission from Getachew et al. [15]

The MediSens (Los Angeles, CA) handgrip, a research device developed at the Wireless Health Institute of the University of California Los Angeles, was used as a sensing platform to measure grip strength in real-time [15]. The device, which is illustrated in Fig. 1, consists of a handle, springs, and a displacement sensor embedded in the frame. The displacement sensor detects the position of the handle, and Hooke’s law (*F* = − *k* ⋅ *x*) is used to convert the position to the grip fosrce. The force is transmitted to a laptop computer where it provides visual feedback during task performance.

During testing, each subject was seated facing a laptop computer with his/her forearm supported on a table and the dominant hand grasping the handle of the device in the lateral position (with the thumb on top). Subjects were positioned such that they were able to keep their hand on the table and the device independently, without need for attachment. On a weekly basis, each subject’s maximum voluntary contraction (MVC) was determined by having the subject grip the handle with maximum strength. Given this was a longitudinal study, frequency of the intervention was limited to once a week in an attempt to optimize subject compliance. Tracking tasks were calibrated to the measured MVC such that the maximum amplitude of the graph shown in Fig. 1 was equal to each subject’s MVC. Each subject was then instructed to vary his/her grip strength according to a sinusoidal (sine) pattern (Fig. 1) that moved across the screen at a constant speed. A grip force indicator displayed the patient’s grip strength in real time. The objective of the test was to minimize the distance between the grip force indicator and the track. The duration of each trial was 45 s, and each subject completed three trials per clinical encounter with approximately five minutes to rest between each trial. The mean absolute error (MAE), which is the average distance between the target waveform and the patient’s response, was used to quantify performance. MAE is frequently applied to target-tracking tasks and effectively measures sensory-motor control capacity [16]. Mean absolute accuracy (MAA), which is equal to (1 – MAE) × 100, was calculated to report performance scores.

The Spinal Cord Independence Measure (SCIM) was used to measure each subject’s independence prior to and after the testing protocol. SCIM is a 19-item instrument that assesses three domains: self-care, respiration and sphincter management, and mobility [17]. The scores range from 0 to 100, and a higher score indicates greater independence.

### Data analysis

Trends in MVC and sine tracking task performance over time were evaluated with the coefficient of determination (r^2^). The r^2^ and associated *p* values were generated with a previously published application that analyzes trends for statistical meaningfulness [18]. Mann-Whitney *U* test was used to compare the cohort’s initial and final MVC and MAA values.

## Results

One subject was lost from the study due to transportation difficulties. A total of 17 subjects (10 males, 7 females) with an average age of 31.3 years (range: 20 – 60) were included in the study results. The subjects’ ASIA grades included A (12), B (1), C (2), and D (2). The levels of SCI included C1 - C2 (1), C3 - C4 (2), C4 - C5 (4), C5 - C6 (7), and C6 - C7 (3). Additional demographic data are shown in Table 1. The average time since injury was 91 months. The average duration of participation in the study was 21.3 weeks (range: 16 – 25).

**Table 1:** Cohort demographics

Patient no.	Gender	Age	AIS	Injury level	Time since injury (months)	Injury mechanism
1	F	22	A	C3-4	75	MVA
2	M	20	B	C6-7	70	GSW
3	M	28	A	C5-6	60	Diving
4	F	24	A	C4-5	62	MVA
5	M	25	A	C6-7	71	GSW
6	F	20	A	C4-5	55	MVA
7	M	38	A	C6-7	180	MVA
8	M	26	A	C5	54	MVA
9	F	44	D	C5-6	114	MVA
10	F	33	D	C5	69	MVA
11	M	24	C	C5	111	Diving
12	M	60	A	C3-4	63	MVA
13	F	20	A	C4-5	122	MVA
14	M	60	A	C5-6	60	MVA
15	M	30	A	C1-2	261	MVA
16	F	25	A	C5-6	64	MVA
17	M	21	C	C4-5	57	MVA

*AIS* American Spinal Injury Association Impairment Scale, *MVA* Motor vehicle accident, *GSW* Gunshot wound

**Fig. 2:**
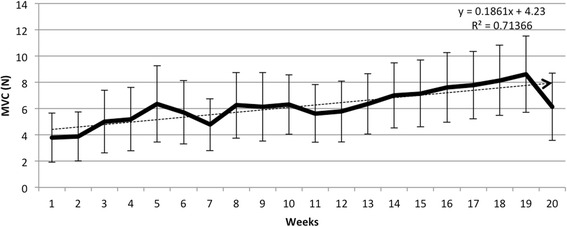
Change in average maximum voluntary contraction with standard error over time for the cohort

As shown in Fig. 2, the initial mean MVC for the entire cohort was 3.78 N. This increased to 6.14 N at week 20, which was not statistically significant (*p* = 0.42). The cohort’s average MVC scores did have a positive correlation with time, however (r^2^ = 0.71). Absolute MVC values were higher in the four motor incomplete subjects and demonstrated greater improvement, with average MVC increasing from 4.1 N to 21.2 N over 20 weeks. In subgroup analyses of the motor complete and motor incomplete groups, neither experienced significant improvements in MVC. Out of the entire cohort, subject #10 demonstrated the greatest absolute improvement of 14.2 N. The average number of sessions to obtain an improvement in MVC was 6.73 for motor incomplete patients and 1 for motor complete patients. This approached but did not meet statistical significance (*p* = 0.07).

**Fig. 3:**
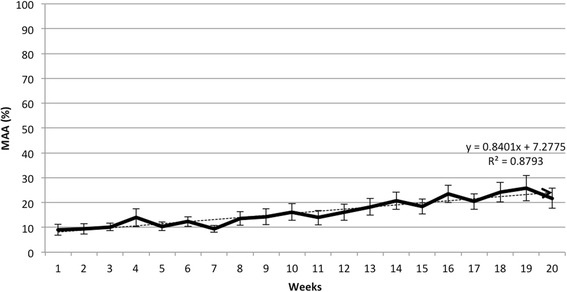
Change in average mean absolute accuracy (MAA) with standard error on the sinusoidal tracking task over time for the cohort

Performance on the sine tracking task is shown in Fig. 3. The average MAA for the cohort increased from 9.01% at week one to 21.7% at week 20. This increase was statistically significant (*p* = 0.02). The cohort’s average MAA scores showed a strong correlation with time (r^2^ = 0.88). In a subgroup analysis, the average initial MAA for motor complete subjects was 5.7%, and at the time of each subject’s final session, this increased to 20.7% (*p* = .001). The average MAA for motor incomplete subjects also increased significantly from 12.8% at the beginning of the study to 35.1% (*p* = 0.04). Out of the entire cohort, subject #11 demonstrated the largest absolute increase in MAA of 42.2%. There was no significant difference between the mean number of sessions required to yield an improvement in MAA between motor complete patients (5.46 sessions) and motor incomplete patients (5.25 sessions).

**Fig. 4:**
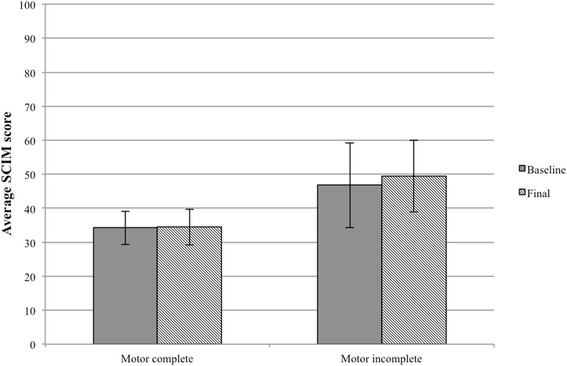
Average Spinal Cord Independence Measure (SCIM) scores with standard error at the beginning and end of the study for each group

At week 1, the average SCIM score of the cohort was 37.18. By the end of the study, the cohort’s average SCIM score was unchanged at 38.0. In a subgroup analysis, neither the motor incomplete or motor complete patients had experienced significant improvements in SCIM score (Fig. 4). Patient #17 had the greatest absolute improvement in SCIM score of 20 points.

## Discussion

Rehabilitation of hand function is an important goal for patients with chronic SCI that has implications for improving QoL, social participation, and functional independence. SCI patients frequently rank improvement in hand and arm function as their priority, above walking and bowel or bladder function [19, 20]. This pilot study was performed to determine if ABT with a novel handgrip device would be feasible and efficacious in patients with chronic SCI. The handgrip was used to administer standardized ABT and to obtain quantitative feedback regarding each patient’s performance. This device has not been used in SCI patients before. Other mechanical adjuncts to upper extremity ABT that have been described in SCI patients typically address the arm and hand together. These include robotic orthoses that assist with task performance [21–23]. These have shown mixed efficacy in regard to their therapeutic potential [23]. Our device is unique in that it is tailored to isolate grip modulation, which is a fundamental skill ubiquitous in ADLs.

Published exercise and activity-based interventions have shown mixed effects on upper extremity function in chronic SCI. Significant improvement in upper extremity strength was reported by Hicks et al. with a twice weekly standardized arm exercise protocol [24]. Conversely, Glinsky et al. did not find improvement in wrist strength after a thrice weekly exercise program, but their intervention duration was only eight weeks [25]. Similar to our results, multiple studies have failed to show improvement in grip strength among motor incomplete subjects after upper extremity ABT [23, 26]. Although the MVC of the cohort trended up over the course of the study, no significant improvement was observed. Given the variability of reported results, further investigation into the effects of ABT on this domain is warranted. Since gripping is an integral part of the ADLs, it should be included in rehabilitation for SCI and quantitative feedback should be employed to realize improvement in this important activity. The MediSens handgrip device was able to standardize the delivery and assessment of this task.

Task performance is an important component of rehabilitation for SCI [8], and we measured this with the handgrip-based sine tracking task. We chose this activity because it requires coordinated manipulation of grip strength (including active muscle contraction and relaxation) that is abundant in the ADLs. Moreover, this test has previously shown to be feasible and efficacious in measuring fine motor function of patients with other neurologic disorders [15, 27]. We observed a significant improvement in accuracy on our handgrip-based tracking task over time. Although tracking tasks have not been used in rehabilitation for SCI before, the Graded Redefined Assessment of Strength, Sensibility, and Prehension (GRASSP) involves grasping tasks and has been used to evaluate SCI subjects’ responses to therapy [28]. Zariffa et al. noted improvements in the GRASSP prehension subscore among motor incomplete subjects engaging in robotic-assisted or non-assisted upper limb rehabilitation [23]. This corroborates our finding that AIS C and D subjects can improve hand task performance with repeated practice. Our study demonstrates that significant improvements can be realized in subjects with motor complete injuries as well. Therefore, it should be a component of rehabilitation for all severities of SCI.

While noticeable improvement in task performance was observed, this did not translate to an increase in overall independence. Small clinically significant improvement in the SCIM score has been defined as four points, and substantial clinical improvement has been defined as ten points on the SCIM scale [29]. Other studies also failed to show significant improvements in independence after ABT [30]. This is unexpected, since SCIM scores correlate well with assessments of hand and arm motor strength and capacity [31–33]. The lack of improvement in grip strength in our group may have prevented the development of greater independence. Regardless, independence is a complex measure that takes into account other domains such as mobility, transfers, respiration, and sphincter management, which all need to be addressed in neurorehabilitation for SCI. The discrepancy between changes in hand function and independence in our study emphasizes the importance of comprehensive, multi-modal therapy to address function of the entire arm. A recent systematic review demonstrated greater improvements in motor function among those patients undergoing multi-modal therapy with a rehabilitation component rather than single interventional approaches [34]. Of these modalities, neuromodulatory techniques such as transcutaneous stimulation have shown promise in improving motor control [35, 36]. Epidural stimulation has also been shown to enable volitional hand movements similar to the ones tested in this study in two tetraplegic patients in a recent proof-of-concept study [37].

The physiologic mechanism by which hand task performance improves with repeated practice is unclear. Repeated use of the handgrip may provide sensory input to the cortex that stimulates neural plasticity. This process facilitates reorganization of relevant descending subcortical pathways that are not damaged. In a cohort of rats subjected to corticospinal and rubrospinal tract injuries randomized to daily reaching and grasping rehabilitation, this group experienced higher degree of recovery of these tasks [38]. Histologically, the density of reticulospinal processes was greater in normal and ectopic areas of ventral grey matter in caudal levels of the spinal cord [36]. Cortical reorganization has been implicated as well [39].

The ABT protocol that we have described was found to be feasible. Participation in our program only required weekly attendance, which is a reasonable time commitment for the study population. Previous recommendations included thrice weekly therapy, which was reduced after the demonstration of improvement following twice weekly participation [21]. The duration of therapy was an average of 20 weeks, which is less than several other published ABT and exercise-based interventions (6 months [6, 7], 9 months [21], and 18 months [28]). The only shorter, non-robotic program that we identified was 8 weeks and did not have an effect on outcome [25]. The equipment required for the MVC and sine tracking tasks is simple for subjects to operate, portable, and provides instantaneous, quantitative feedback about performance. Compared to robotic devices used for rehabilitation, the handgrip is simpler to operate and requires minimal supervision.

Generalization of the findings in this study is limited by the small sample size. The fact that only one patient was lost from the study suggests SCI patients are able to engage in handgrip testing and tolerate repeated evaluations. Despite our small cohort, we were able to identify a significant improvement in task performance among both motor complete and motor incomplete subjects. A larger cohort is needed to determine the true extent of improvement and to identify subsets of patients who are most likely to benefit. This study was also limited by the fact that the speed of the tracking task has not previously been validated, and older patients’ performances may have been limited by age-related deficits in reaction time. Regardless, similar tracking tasks have been applied to older populations with reliable results [40].

## Conclusion

A weekly activity-based therapy program involving repeated hand contraction and task performance with a novel handgrip device is feasible and improves hand task performance. Evaluation with a larger cohort is merited.

## Abbreviations

ABT: Activity-based therapy
ADL: Activity of daily living
AIS: American Spinal Injury Association Impairment Scale
ASIA: American Spinal Injury Association
GRASSP: Graded Redefined Assessment of Strength, Sensibility, and Prehension
MAA: Mean absolute accuracy
MAE: Mean absolute error
MVC: Maximum voluntary contraction
QoL: Quality of life
SCI: Spinal cord injury
SCIM: Spinal cord independence measure
